# Supplementary material for: The Role of Cellular Senescence in Cyclophosphamide-Induced Primary Ovarian Insufficiency
Source: Int J Mol Sci. 2023 Dec 6;24(24):17193. doi: 10.3390/ijms242417193 (PMC10743614; doi:10.3390/ijms242417193)
Supplement: Supplementary file 1 [file ijms-24-17193-s001.zip › ijms-2720664-supplementary.pdf]

Supplementary Information

The role of cellular senescence in cyclophosphamide-induced primary ovarian insufficiency

Zixin Xu et al.

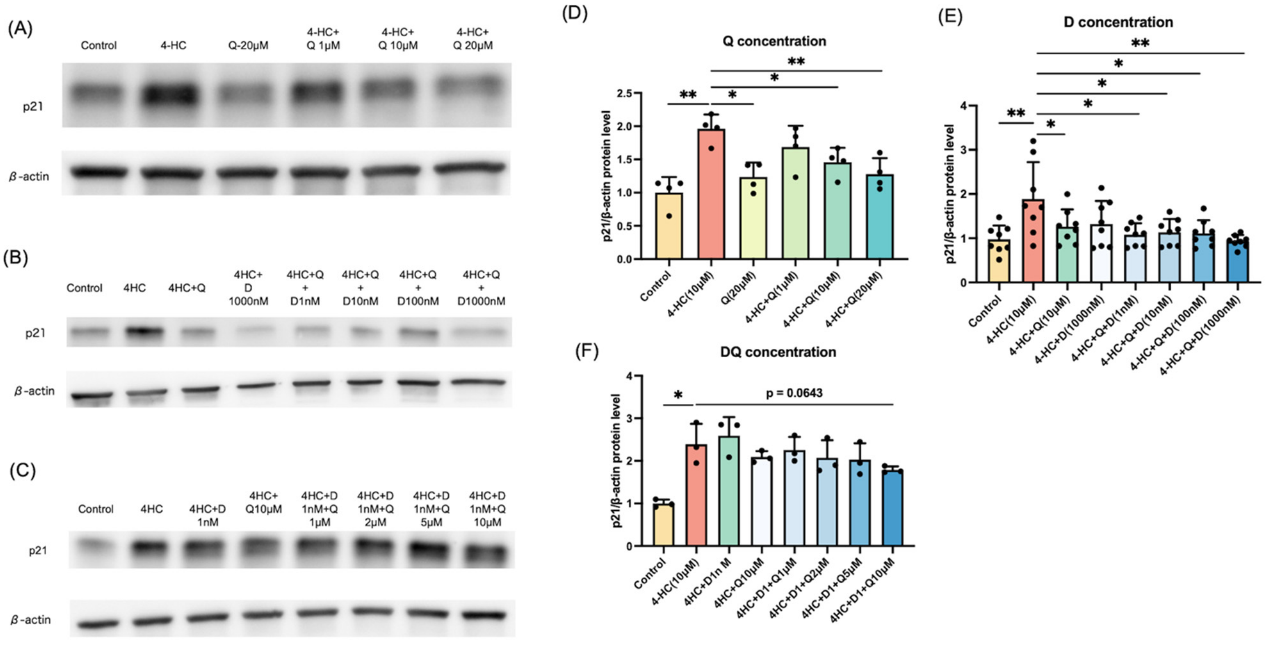

Supplementary Figure S1. Determination of the optimal concentrations of DQ. (**A, D**) Human GLCs were pretreated with 1, 10, and 20 μM quercetin for 24 h before administration of 4-HC. (**B, E**) Human GLCs were pretreated with 10 μM quercetin and 1, 10, 100, and 1000 nM dasatinib for 24 h before administration of 4-HC. (**C, F**) Human GLCs were pretreated with 1 nM dasatinib and 1, 2, 5, and 10 μM quercetin for 24 h before administration of 4-HC. Protein expression of p21 in human GLCs was analyzed by western blotting. β-actin was used as a loading control. Representative images are shown in A–C. Quantitative analysis of western blotting is shown in D–F. Values represent mean ± SD. Student's *t*-test was used for statistical analysis. * $p < 0.05$ and ** $p < 0.01$ compared with the 4-HC-treated group. D, dasatinib; Q, quercetin.
